# Aortic Pseudoaneurysm following Ventricular Septal Defect Closure in a Pediatric Patient: A Case Report and Literature Review

**DOI:** 10.1155/2023/2890844

**Published:** 2023-11-23

**Authors:** Hassan Zamani, Kourosh Vahidshahi, Mohammad Reza Khalilian, Tahmineh Tahouri, Ehsanollah Rahimi-Movaghar

**Affiliations:** ^1^Shahid Modarres Educational Hospital, School of Medicine, Shahid Beheshti University of Medical Sciences, Tehran, Iran; ^2^Department of Paediatrics, Shahid Beheshti University of Medical Science, Tehran, Iran; ^3^Shahid Modarres Educational Hospital, Shahid Beheshti University of Medical Sciences, Tehran, Iran; ^4^Department of Surgery, Farhikhtegan Hospital, Faculty of Medicine, Tehran Medical Science, Islamic Azad University, Tehran, Iran

## Abstract

Aortic pseudoaneurysm, a rare condition characterized by localized transmural disruption and dilatation of the aorta, is very rare in the pediatric population. It is primarily caused by previous cardiovascular procedures such as aortic coarctation repair, aortic valve replacement, and subaortic membrane resection. We present a unique case of aortic pseudoaneurysm following surgery to repair a perimembranous ventricular septal defect in a 19-month-old boy who presented with fever as the sole symptom. The fever started on the 30th day after the surgery, and the patient exhibited abnormal laboratory results, including a white blood cell (WBC) count of 28.3 × 109/L, neutrophil percentage of 68%, platelet count of 880 × 109/L, erythrocyte sedimentation rate (ESR) of 200 mm/hour, and 3+ positive C-reactive protein. Echocardiogram revealed a large cystic mass (5 × 4.8 cm) in the ascending aorta, compressing the superior vena cava. Based on this finding, a diagnosis of aortic pseudoaneurysm was suspected. The diagnosis was confirmed through cardiac computed tomographic angiography, and the patient underwent emergent surgery for the repair of the aortic pseudoaneurysm under deep hypothermia and circulatory arrest. Unfortunately, our patient died shortly after the surgery.

## 1. Introduction

Aortic pseudoaneurysm, a localized transmural disruption and dilatation of the aortic wall, is very rare in the pediatric population [1]. In most cases, it arises as a rare complication following cardiac surgery, although other causes such as trauma, infection, and genetic disorders can also contribute [2].

## 2. Case Report

Our patient was a 19-month-old boy, who underwent surgery to repair a perimembranous ventricular septal defect (VSD). The VSD was diagnosed shortly after birth, and the patient had regular follow-ups. During the most recent echocardiograms, mild aortic insufficiency due to the right coronary cusp prolapse was observed. Consequently, a decision was made to repair the ventricular septal defect. The patient had no relevant familial history and was otherwise healthy, with an admission weight of 12 kg. Physical examination revealed a 3/6 degree holosystolic murmur in the left sternal border, while examinations of other body systems were normal. The echocardiogram showed a 5 mm perimembranous VSD with right coronary cusp prolapse and mild aortic insufficiency. On the third day of hospitalization, VSD closure under cardiopulmonary bypass (CPB) with median sternotomy was performed. The postoperative course was uneventful, except for mild pericardial effusion which resolved with medical treatment. The patient was discharged on the seventh postoperative day with captopril and furosemide. A follow-up echocardiogram at two weeks postoperatively demonstrated trivial aortic insufficiency, a small residual ventricular septal defect, and mild right ventricular dysfunction. There was no pericardial effusion, and the left ventricular ejection fraction was normal.

One month after the surgery, the patient was readmitted to another center due to a low-grade intermittent fever lasting one week. No signs of pneumonia, gastroenteritis, or urinary tract infection were observed during readmission. The chest X-ray was normal, and other investigations failed to determine the cause of the fever. The patient was discharged after receiving antibiotics for seven days.

**Figure 1:**
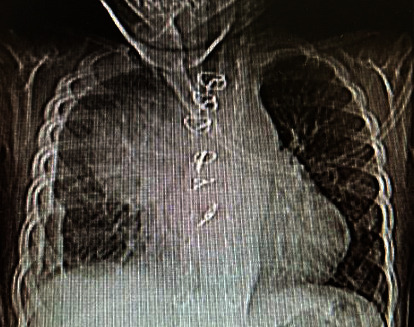
Chest X-ray in AP view showed a large mass in the right superior border.

**Figure 2:**
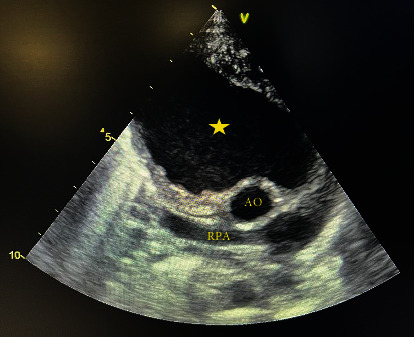
Transthoracic echocardiograph in suprasternal view showed a large pseudoaneurysm arising from the anterior wall of the ascending aorta. RPA: right pulmonary artery; AO: aorta.

**Figure 3:**
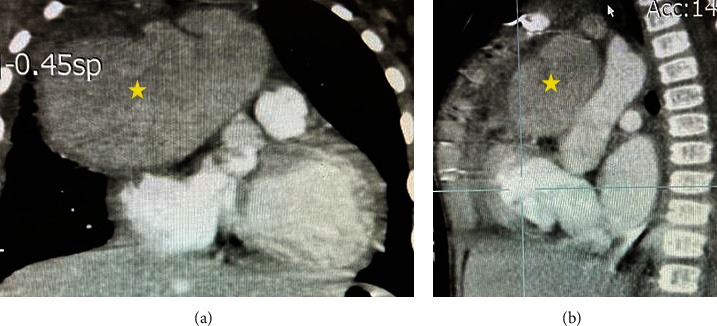
Coronal (a) and sagittal (b) views of contrast enhancement CT image show a large pseudoaneurysm arising from the ascending aorta (asterisks).

A week after being discharged, the patient was referred to our center with a high-grade fever of 39.5°C as the only complaint. At admission, the blood pressure was 95/55 mmHg, and the pulse rate was 108 beats per minute. The patient had no respiratory distress, and heart auscultation revealed a 2/6 grade holosystolic murmur in the left sternal border. Physical examinations of other body systems were normal. The electrocardiogram showed sinus tachycardia, but no other abnormalities were detected. Laboratory investigations revealed a white blood cell (WBC) count of 28.3 × 109/L, neutrophil percentage of 68%, platelet count of 880 × 109/L, erythrocyte sedimentation rate (ESR) of 200 mm/hour, and positive 3+ C-reactive protein. A plain chest X-ray revealed a large mass in the right hemithorax (Figure 1). Transthoracic echocardiography showed a large cystic lesion measuring 5 × 4.2 cm at the anterior aspect of the ascending aorta (Figure 2), compressing the superior vena cava. Cardiac computed tomography angiography (CTA) confirmed the diagnosis of aortic pseudoaneurysm, revealing a large hypoechoic mass (6.2 × 4.3 cm) extending to the right and superior of the ascending aorta, with direct communication between the mass and the aorta (Figure 3).

Subsequently, the patient underwent emergent surgery. Cooling was initiated, and the right femoral artery was cannulated. Median sternotomy was performed after reaching a core temperature of 28°C, followed by venous cannula insertion and initiation of cardiopulmonary bypass. The pseudoaneurysm sac was opened, revealing active bleeding from the aorta. Cooling continued until a core temperature of 18°C was reached, and total circulatory arrest was performed. A 1 cm rupture in the aortic root, at the site of a previous cardioplegic injection, was repaired with a pedicardial patch, with BioGlue applied on the patch. Rewarming and heart function restoration were performed, although the patient experienced frequent episodes of ventricular tachycardia, which were controlled with cardioversion. The patient was weaned from cardiopulmonary bypass (CPB) while receiving epinephrine (0.15 mcg/kg/min) and norepinephrine (0.1 mcg/kg/min). The sternum was left open, and the patient was transferred to the pediatric intensive care unit (ICU). Unfortunately, shortly after being transferred to the ICU, the patient suffered a cardiac arrest. Despite immediate cardiopulmonary resuscitation, it was unsuccessful, and the patient passed away. All blood cultures, both pre- and post-operative, were negative. Pathology examination of the excised vessel revealed granulation tissue formation, foreign body reaction, fibrin deposition, and abscess formation.

## 3. Discussion

Aortic pseudoaneurysm is primarily caused by previous cardiovascular procedures, especially the repair of aortic coarctation [2]. To the best of our knowledge, this may be the third reported case of aortic pseudoaneurysm following isolated ventricular septal defect repair surgery in a child. The two other cases were a 4-year-old girl [3] and a seven-month-old infant [4]. The other reported causes of aortic pseudoaneurysms in the pediatric population include trauma [2, 5], previous aortic valve replacement surgery, and repair of subaortic membrane [6]. Guruchandrasekar et al. also reported spontaneous aortic pseudoaneurysm in an 8-month-old infant with the tetralogy of Fallot [1]. In a case series reported by Malvindi et al., the most common procedures leading to aortic pseudoaneurysm in 27 adult patients were the Bental procedure, ascending aorta repair, aortic valve replacement, mitral valve replacement, David's reimplantation, and coronary artery bypass graft. None of their cases had a history of previous ventricular septal defect repair [7].

Notably, in the current case, pseudoaneurysm occurred at the cardioplegic injection site. Aortic pseudoaneurysm typically occurs at injured sites in the aorta, such as sites of cardioplegic solution injection and needle insertions for pressure measurements [3, 4].

Symptoms of aortic pseudoaneurysm are usually nonspecific, and the condition often remains asymptomatic, necessitating a high degree of clinical suspection for diagnosis [2]. Köner et al. reported a case of aortic pseudo aneurysm presenting with dyspnea and intercostal retraction two months after ventricular septal defect repair [4]. Li et al. described a case of ascending aorta pseudoaneurysm in a 4-year-old girl who presented with fever on the 18th postoperative day [3]. Atiyah et al. reported aortic pseudoaneurysm diagnosed with fever, three weeks after surgery for the repair of ventricular septal defect repair and pulmonary artery debanding in a two-year-old child with a history of bicuspid aortic valve, aortic arch repair, and pulmonary artery banding in the neonatal period [2]. Similar to these cases, our patient also presented with fever as the only symptom.

History and physical examination often yield nonspecific findings. Chest X-ray is generally not sensitive or specific for diagnosis. Echocardiography, a noninvasive, inexpensive, and widely available modality, is useful for the diagnosis of aortic pseudoaneurysm and does not expose the patient to radiation or ionizing contrast. Two-dimensional, color Doppler and pulse wave Doppler echocardiography are all valuable tools. However, while echocardiography serves as a good screening modality, it is not considered as the gold standard for diagnosing aortic pseudoenurysm [8]. Cardiac CT-angiography can confirm the diagnosis, determine the size and extension of the pseudoaneurysm, and detect the presence of hemorrhage (mediastinal or pericardial) [2, 6, 9]. However, the main limitation of computed tomography is exposure to ionizing radiation. MRI is also a sensitive and specific imaging tool for diagnosing aortic pseudoaneurysm [8].

Once the diagnosis of aortic pseudoaneurysm is confirmed, surgery is indicated since spontaneous bleeding complications can be fatal. However, some surgeons may prefer to perform surgery after a course of antibiotic treatment [10]. Standard treatment for aortic pseudoaneurysm in children has not been clearly defined, but surgical repair and interventional approaches are the main options. Interventional repair using atrial septal occlusion devices and endovascular grafts has been reported [11], but surgery remains the gold standard treatment for the pediatric population [1, 2]. In cases where the pseudoaneurysm is located on the aortic arch or ascending aorta, median sternotomy is preferred. Auxiliary right anterior thoracotomy or a minimally invasive “J” incision is the incision of choice when the pseudoaneurysm is in close proximity to the undersurface of the sternum. Aortic graft replacement is the preferred treatment for most patients, although smaller defects can be repaired without using a tubular graft. Extensive debridement of necrotic and infected tissue and the use of allograft conduit are indicated in patients with endocarditis [12].

Surgical repair of an aortic pseudoaneurysm presents various challenges, including hemodynamic compromise due to bleeding, adhesions from previous cardiac surgery, and the risk of cerebral complications from air embolism [2]. Consequently, repairing an aortic pseudoaneurysm is often associated with significant morbidity and mortality. Mortality rates are particularly higher when the pseudoaneurysm is located close to the undersurface of the sternum [12]. Hemorrhage during surgical maneuvers for repairing aortic pseudoaneurysms is the most common cause of mortality during surgery [13].

## 4. Conclusions

Aortic pseudoaneurysm is a rare but life-threatening complication of cardiac surgery, commonly observed at injured sites of the aorta, including the areas where the cardioplegic solutions are injected, and prior surgical sites in the aorta.

## Data Availability

The data that support the findings of this study are available from the corresponding author (T.T.) upon reasonable request.

## References

[1] Guruchandrasekar S. H., Mallula K. K., Siwik E. (2020). Endovascular repair of thoracic aortic pseudoaneurysms in children. *Case Reports*.

[2] Atiyah M., Mohsin S., Al Faraidi L., Al-Hawri K., Al Otay A., Al N. K. (2018). Surgical management of ascending aortic pseudoaneurysm in a 2-year-old boy: a case report. *Journal of Medical Case Reports*.

[3] Li X., Zhou H., Zhang R. (2021). Case report: ascending aortic pseudo-aneurysm following ventricular septal defect repair in a 4-year-old girl. *Frontiers in Pediatrics*.

[4] Köner Ö., Çetin G., Özkara A., Saltık L. (2004). Pseudoaneurysm of the ascending aorta in a pediatric patient. *Turk Gogus Kalp Dama*.

[5] Wang K., Xu W., Wang C., Hu J., Tan S., Fan X. (2023). Aortic repair through a left thoracotomy in a 12-year-old child with traumatic aortic Pseudoaneurysm: a case report. *The Heart Surgery Forum*.

[6] Marcano L., Naranjo A., Diaz F., González A. (2011). Ascending aortic pseudoaneurysm after aortic valve replacement with root enlargement in a pediatric patient. *Pediatric Cardiology*.

[7] Malvindi P. G., Cappai A., Raffa G. M. (2013). Analysis of postsurgical aortic false aneurysm in 27 patients. *Texas Heart Institute Journal*.

[8] Chu L. C., Cameron D. E., Johnson P. T., Fishman E. K. (2012). MDCT evaluation of postoperative aortic root pseudoaneurysms: imaging pearls and pitfalls. *American Journal of Roentgenology*.

[9] Barth H., Moosdorf R., Bauer J., Schranz D., Akintürk H. (2000). Mycotic pseudoaneurysm of the aorta in children. *Pediatric Cardiology*.

[10] Alhawri K. A., Elsaedy U. H., Alahdal S. A., Albahlooli N. S., Al Qwaee A. A., Alakhfash A. A. (2020). Lethal recurrent mycotic ascending aortic pseudoaneurysm in a 21-month-old child with repaired subaortic membrane. *Annals of Pediatric Cardiology*.

[11] Stamou S. C., Conway B. D., Nores M. A. (2020). Management of aortic pseudoaneurysms: evolving concepts and controversies. *Aorta*.

[12] Atik F. A., Navia J. L., Svensson L. G. (2006). Surgical treatment of pseudoaneurysm of the thoracic aorta. *The Journal of Thoracic and Cardiovascular Surgery*.

[13] Vahedian J., Sadeghpour A. (2006). Arterial homograft and medical therapy in pseudoaneurysm of infrarenal aorta concomitant with recurrent right ventricular thrombus in Behcet's disease. *Saudi Medical Journal*.

